# Plasma CD55 and HIV RNA load in newly diagnosed antiretroviral therapy-naive adults: an exploratory hospital-based case-control study

**DOI:** 10.3389/fimmu.2026.1981826

**Published:** 2026-10-20

**Authors:** Lizhong Li, Zhe Hou, Hanping Li, Jian Wang, Wei Zhang, Jing Xie, Huan Li, Yuxin Feng, Ziwei Liu, Lin Li, Leili Jia

**Affiliations:** 1 Chinese PLA Center for Disease Control and Prevention, Beijing, China; 2 Dezhou Center for Disease Control and Prevention, Dezhou, China; 3 State Key Laboratory of Pathogen and Biosecurity, Academy of Military Medical Sciences, PLA Academy of Military Science, Beijing, China

**Keywords:** alternative pathway, case-control study, complement activation, complement regulation, HIV, HIV RNA load, plasma CD55

## Abstract

**Background:**

Plasma complement regulation during untreated HIV infection remains incompletely characterized. We examined complement markers in newly diagnosed, antiretroviral therapy (ART)-naive adults and their relationships with HIV RNA load.

**Methods:**

Archived EDTA plasma from 132 HIV-positive adults and 59 HIV-negative controls was analyzed for eight complement markers by ELISA/EIA. Case-control models were adjusted for age and sex, with false discovery rate control across eight markers. Within HIV-positive participants, analyses compared four viral-load categories and modeled log10 HIV RNA load with adjustment for age, sex, and CD4+ T-cell count.

**Results:**

All eight markers had higher unadjusted concentrations in HIV-positive participants than in controls. Seven case-control associations remained significant after adjustment and false discovery rate correction; CD59 did not. CD55 differed across viral-load categories (<10³, 10³ to <10^4^, 10^4^ to <10^5^, and ≥10^5^ copies/mL), with higher concentrations in the highest than the lowest category. Higher HIV RNA load was associated with higher Ba and properdin (complement factor P, CFP) concentrations after eight-marker correction. For CD55, β was 0.067 (95% CI 0.008 to 0.125; nominal P = 0.0260). The CD55 association did not remain significant after correction across eight markers (q=0.0693).

**Conclusion:**

Untreated HIV infection was accompanied by differences in plasma complement activation fragments and regulatory proteins. The CD55–HIV RNA load association was not supported consistently across analyses and requires independent validation.

## 1 Introduction

Human immunodeficiency virus (HIV) infection is marked by CD4+ T-cell loss, persistent immune activation, and inflammatory signaling, including before antiretroviral therapy (ART) is started (1, 2). Plasma markers measured near diagnosis may provide an indirect view of host pathways accompanying active viral replication.

Complement is one such pathway. It links pathogen recognition to opsonization, inflammatory amplification, and effector injury, while being restrained by soluble and membrane-associated regulators. The classical, lectin, and alternative pathways converge on C3/C5 convertase formation, but markers sampled at different points in the cascade reflect different aspects of the pathway. Here, C1q and MASP-2 were treated as classical- and lectin-pathway-associated proteins, respectively; Ba as an alternative-pathway activation product; C3a and C5a as downstream activation fragments; and CD55, CD59, and properdin (complement factor P, CFP) as regulatory or amplification-related proteins (3–5).

HIV makes this balance especially difficult to interpret. The virus can activate complement through envelope-associated interactions with C1-related components (6, 7), yet virions can also incorporate or exploit host regulators, including CD55, CD59, and CD46, to reduce complement-mediated destruction (8, 9). Complement-opsonized HIV can influence immune sensing and cell-virus interactions, so plasma complement findings in patients cannot be interpreted as a uniform change in complement activity (10–12).

Clinical studies that map HIV replication to a broad plasma complement panel remain relatively uncommon, particularly in untreated participants sampled around first diagnosis. Prior patient studies have largely involved treated cohorts, limited complement readouts, or broader inflammatory and coagulation biomarker panels (13, 14). Mechanistic studies have also relied heavily on *in vitro* models (10–12). We therefore compared eight plasma complement markers between HIV-positive participants and HIV-negative controls recruited from the same hospital testing setting, and examined their associations with HIV RNA load within the HIV-positive group.

## 2 Materials and methods

### 2.1 Study design and setting

This observational case-control study used participant data and archived EDTA plasma collected in 2020 from collaborating hospitals in a North China region. Reporting followed the STROBE framework (15). HIV-positive participants were newly diagnosed through antibody testing; hospital review confirmed that all were ART-naive at sampling. HIV-negative controls were volunteers seeking HIV testing in the same hospital testing setting and study period who tested negative for HIV antibodies. Clinical information and specimens were centralized for complement measurement and analysis.

### 2.2 Participants and eligibility criteria

Eligibility required age ≥18 years, complete core analytical variables, and sufficient archived EDTA plasma for complement measurement. Core variables were age, sex, and CD4+ T-cell count in both groups, plus plasma HIV RNA load in HIV-positive participants. Among 442 HIV-positive candidates, 84 lacked CD4 count or viral-load data, 224 had insufficient plasma volume, and two were aged <18 years; 132 were included. Among 108 HIV-negative candidates, 48 had insufficient plasma volume and one was aged <18 years; 59 were included (**Supplementary Figure S1**). All eligible adult specimens were included; the available specimens determined the sample size. Controls were not prospectively matched to cases by age or sex.

### 2.3 Clinical variables and definitions

CD4+ and CD8+ T-cell counts were determined by single-platform three-color flow cytometry on a BD FACSCanto II flow cytometer. BD Tritest CD4/CD8/CD3 reagent (cat. no. 340298) and BD Trucount absolute counting tubes (cat. no. 340334) were used (BD Biosciences, San Jose, CA, USA). Plasma HIV-1 RNA was quantified by real-time reverse-transcription PCR using the COBAS AmpliPrep/COBAS TaqMan HIV-1 Test v2.0 (P/N 05212294190). The manufacturer was Roche Molecular Systems (Branchburg, NJ, USA), and the linear quantification range was 20–10^7^ copies/mL. The assay targets gag and LTR sequences and quantifies HIV-1 group M and group O, rather than HIV-2 RNA. Viral-load and complement measurements used specimens from the same blood collection. HIV RNA load was categorized as <10³, 10³ to <10^4^, 10^4^ to <10^5^, and ≥10^5^ copies/mL. Tuberculosis and opportunistic infection (OI) status were obtained from available infectious-disease reporting cards, medical records, and clinical history taking. The OI/tuberculosis fields documented infections present at HIV diagnosis; their activity and related treatment at plasma sampling could not be established. Twelve participants had recorded OI, including all three tuberculosis cases. No coinfection was clinically identified in the remaining 120 HIV-positive participants from the available information. These data did not constitute systematic infection screening. Hepatitis B and C testing was not performed for this study.

### 2.4 Plasma collection, storage, and handling

Blood was collected into EDTA anticoagulant tubes. Plasma was separated, aliquoted, and stored at −80 °C for less than one year before complement testing. The aliquots used for ELISA/EIA were thawed before testing and had not undergone any previous freeze-thaw cycle. The same handling procedures were used for both groups.

### 2.5 ELISA quantification of complement markers

Plasma C3a, C5a, C1q, Ba, MASP-2, CD55, CD59, and properdin/CFP were quantified by commercial ELISA/EIA according to the manufacturers’ protocols. Quidel MicroVue assays (San Diego, CA, USA) measured C3a (A031), C5a (A021), and Ba (A033). Hycult Biotech assays (Uden, the Netherlands) measured C1q (HK356), MASP-2 (HK326), and soluble CD59 (HK374-01). CD55 was measured using Sino Biological KIT10101-1 (Beijing, China), and properdin using RayBiotech ELH-PROPE-1 (Peachtree Corners, GA, USA). Optical density was read at 450 nm, and concentrations were calculated from the corresponding standard curves with correction for the applied dilution factors. Assay details and marker- and group-specific dilution factors are listed in **Supplementary Table S1**. CD59 denotes the plasma measurement obtained with the soluble-CD59 assay; neither regulatory-marker assay measured cell-surface expression.

### 2.6 Statistical analysis

Continuous variables were summarized as median [IQR] or mean ± SD and categorical variables as n (%). Unadjusted case-control comparisons used two-sided Mann-Whitney U tests. Kruskal-Wallis tests compared the four HIV-positive viral-load categories; HIV-negative controls were not included. Omnibus P values were unadjusted for multiple testing. For markers with omnibus P<0.05, Dunn tests with tie correction and Holm adjustment across six pairwise comparisons were used. Log10 marker concentrations were modeled against HIV status with age and sex adjustment. All regression models used HC3 heteroskedasticity-robust standard errors. Benjamini-Hochberg correction was applied across the eight case-control models. Selected-marker case-only models related log10 CD55, Ba, and properdin/CFP to log10 HIV RNA load, adjusting for age, sex, and CD4+ T-cell count. Their P values were nominal. Extended exploratory case-only analyses included all eight markers, with Benjamini-Hochberg correction across that separate model family.

Sensitivity analyses included age-adjusted male-only models and optimal 1:1 matching without replacement, exact for sex within a five-year age caliper. Matching maximized the number of pairs and then minimized total absolute age distance; implementation details are provided in **Supplementary Table S5**. Matched log10 concentration differences were evaluated using one-sample t tests with 95% confidence intervals. Restricted analyses excluded the 12 HIV-positive participants with recorded tuberculosis or OI, retaining the same 59 controls. Case-control models, case-only models, and viral-load comparisons were repeated in this population, with multiplicity correction within the respective analysis families. Additional exploratory comparisons used CD4+ T-cell strata (<200, 200–500, and >500 cells/μL) and equal-sized HIV RNA load quartiles. These used the same omnibus and *post hoc* procedure. All tests were two-sided; P<0.05 or the applicable adjusted P or q<0.05 defined statistical significance. Analyses used Python 3.12.14, SciPy 1.17.0, statsmodels 0.14.6, and scikit-posthocs 0.17.0.

### 2.7 Ethics and consent

The study involving human participants was reviewed and approved by the Ethics Committee of the National Center for STD/AIDS Prevention and Control, Chinese Center for Disease Control and Prevention (approval number: X190311577). Written informed consent for research use of de-identified clinical information and plasma was obtained in accordance with the approved protocol.

## 3 Results

### 3.1 Study participants and baseline characteristics

The final analysis included 191 adults: 132 HIV-positive participants and 59 HIV-negative controls (**Supplementary Figure S1**). Median age was 28.0 years in the HIV-positive group and 40.0 years in controls; 97.0% and 52.5%, respectively, were male. Mean CD4+ T-cell count was 315.13 ± 175.31 cells/μL among HIV-positive participants and 1075.58 ± 271.22 cells/μL among controls. HIV RNA load strata within the HIV-positive group were <10³ copies/mL (n=16), 10³ to <10^4^ (n=30), 10^4^ to <10^5^ (n=63), and ≥10^5^ (n=23). Twelve HIV-positive participants had OI documented at HIV diagnosis, including all three participants with recorded tuberculosis (**Table 1**).

**Table 1:** Baseline characteristics of the study population.

Variable	HIV+ (n=132)	HIV− (n=59)
Age, years (median [IQR])	28.0 [23.0-38.2]	40.0 [32.5-53.0]
Male, n (%)	128 (97.0%)	31 (52.5%)
CD4+ T cells (mean ± SD), cells/μL	315.13 ± 175.31	1075.58 ± 271.22
Recorded tuberculosis, n (%)	3 (2.3%)	0 (0.0%)
Recorded OI, including tuberculosis, n (%)	12 (9.1%)	0 (0.0%)
Viral load strata (HIV+), n (%)	<10³: 16 (12.1%); 10³ to <10^4^: 30 (22.7%); 10^4^ to <10^5^: 63 (47.7%); ≥10^5^: 23 (17.4%)	—

### 3.2 Plasma complement markers differed between HIV-positive participants and controls

Unadjusted comparisons showed higher concentrations of all eight measured complement markers in HIV-positive adults than in HIV-negative controls (**Figure 1**; **Table 2**). Given the differences in age and sex between the groups, these unadjusted findings should be interpreted alongside the age- and sex-adjusted analyses presented in Section 3.4.

**Figure 1:**
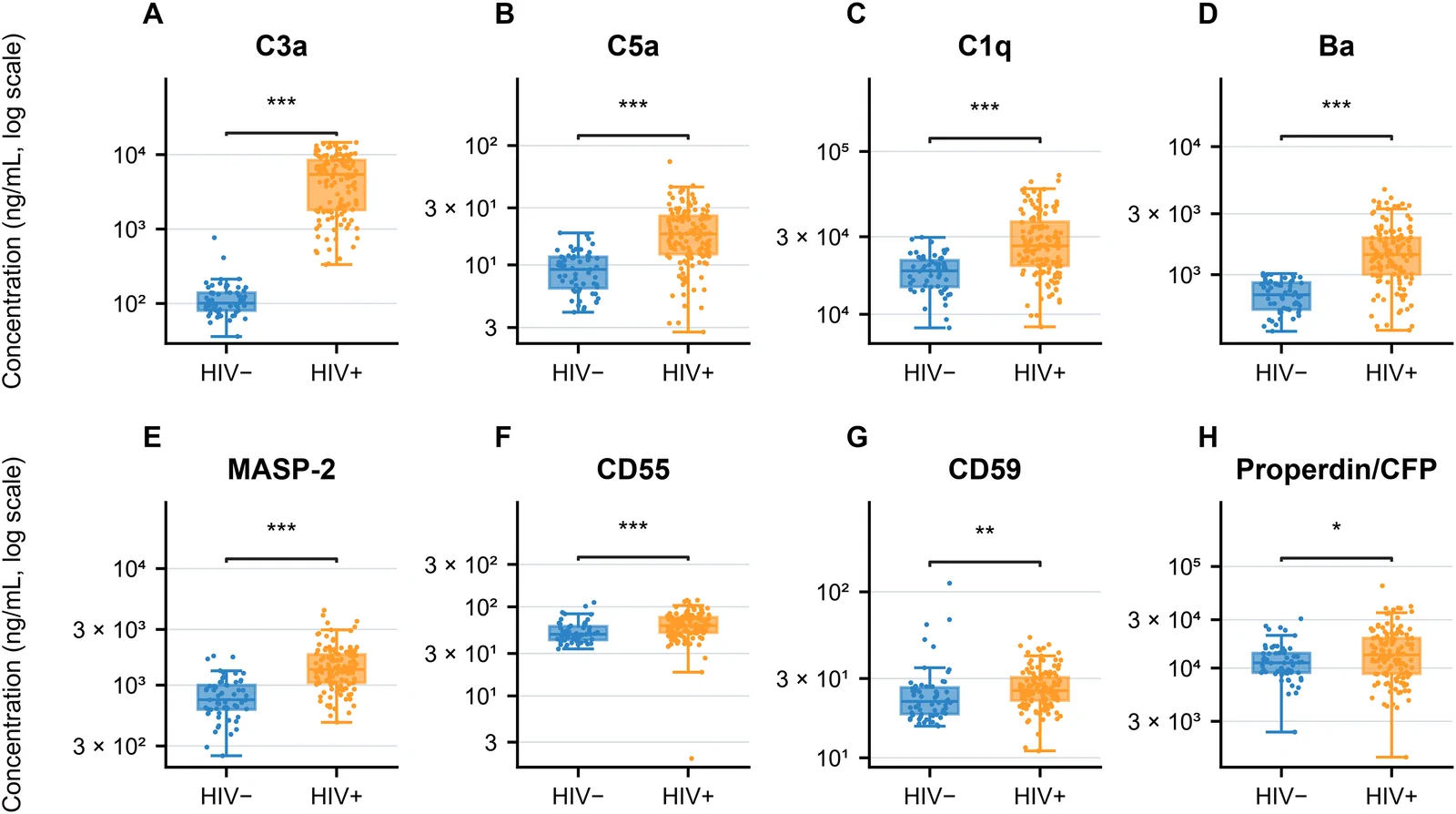
Plasma complement markers in HIV-positive adults and HIV-negative adult controls. **(A–H)** show C3a, C5a, C1q, Ba, MASP-2, CD55, CD59, and properdin/CFP, respectively. Box-and-whisker plots show the median and interquartile range (IQR); whiskers extend to the most extreme observations within 1.5 times the IQR of the lower and upper quartiles, and points represent individual participants. Concentrations are displayed on logarithmic y-axes. Case-control comparisons used unadjusted Mann-Whitney U tests; *P<0.05; **P<0.01; ***P<0.001. Adjusted estimates are shown in **Figure 3** and **Supplementary Table S4**.

**Table 2:** Case-control comparison of plasma complement markers.

Marker (ng/mL)	HIV+ median [IQR]	HIV− median [IQR]	P
C3a	5409.86 [1814.87-8483.62]	100.81 [80.14-138.68]	<0.001
C5a	18.10 [12.28-25.76]	9.15 [6.39-11.63]	<0.001
C1q	26356.07 [19904.47-37076.05]	18526.89 [14811.02-21451.99]	<0.001
Ba	1435.13 [1009.16-1941.28]	697.40 [536.05-864.88]	<0.001
MASP-2	1356.18 [1045.94-1825.13]	745.17 [616.43-996.40]	<0.001
CD55	61.82 [51.28-76.56]	49.07 [42.69-60.23]	<0.001
CD59	25.30 [22.13-30.44]	21.82 [18.38-26.50]	0.0011
Properdin/CFP	13488.42 [8815.32-19664.12]	11269.50 [9021.90-14034.94]	0.044

### 3.3 Complement markers across HIV RNA load strata and in continuous models

Among HIV-positive participants, CD55, Ba, properdin/CFP, and C3a differed across the four viral-load categories (**Table 3**; **Supplementary Table S2**). Dunn-Holm comparisons showed higher CD55 in the ≥10^5^ than the <10³ copies/mL category (adjusted P = 0.0201). Ba was higher in the ≥10^5^ category than in the <10³ and 10³ to <10^4^ categories (adjusted P = 0.0132 and 0.0098). Properdin/CFP was higher in the 10^4^ to <10^5^ and ≥10^5^ categories than in the <10³ category (adjusted P = 0.0286 and 0.0057). Other pairwise comparisons were not significant, including all C3a comparisons. The differences therefore did not establish a stepwise increase between successive viral-load categories (**Figures 2A–C**).

**Table 3:** Omnibus and *post hoc* comparisons among HIV-positive viral-load strata.

Marker	Kruskal-Wallis P	Significant Dunn-Holm comparisons	Adjusted P
C3a	0.0293	None	—
CD55	0.0170	<10³ versus ≥10^5^	0.0201
Ba	0.0024	<10³ versus ≥10^5^ 10³–<10^4^ versus ≥10^5^	0.0132 0.0098
Properdin/CFP	0.0097	<10³ versus 10^4^–<10^5^ <10³ versus ≥10^5^	0.0286 0.0057

HIV-positive participants only (n=132). HIV-negative controls were excluded from all tests in this table. Omnibus P values are unadjusted; Dunn P values are Holm-adjusted across six comparisons per marker. Complete concentrations and comparisons appear in **Supplementary Table S2**. Viral-load categories are in copies/mL.

**Figure 2:**
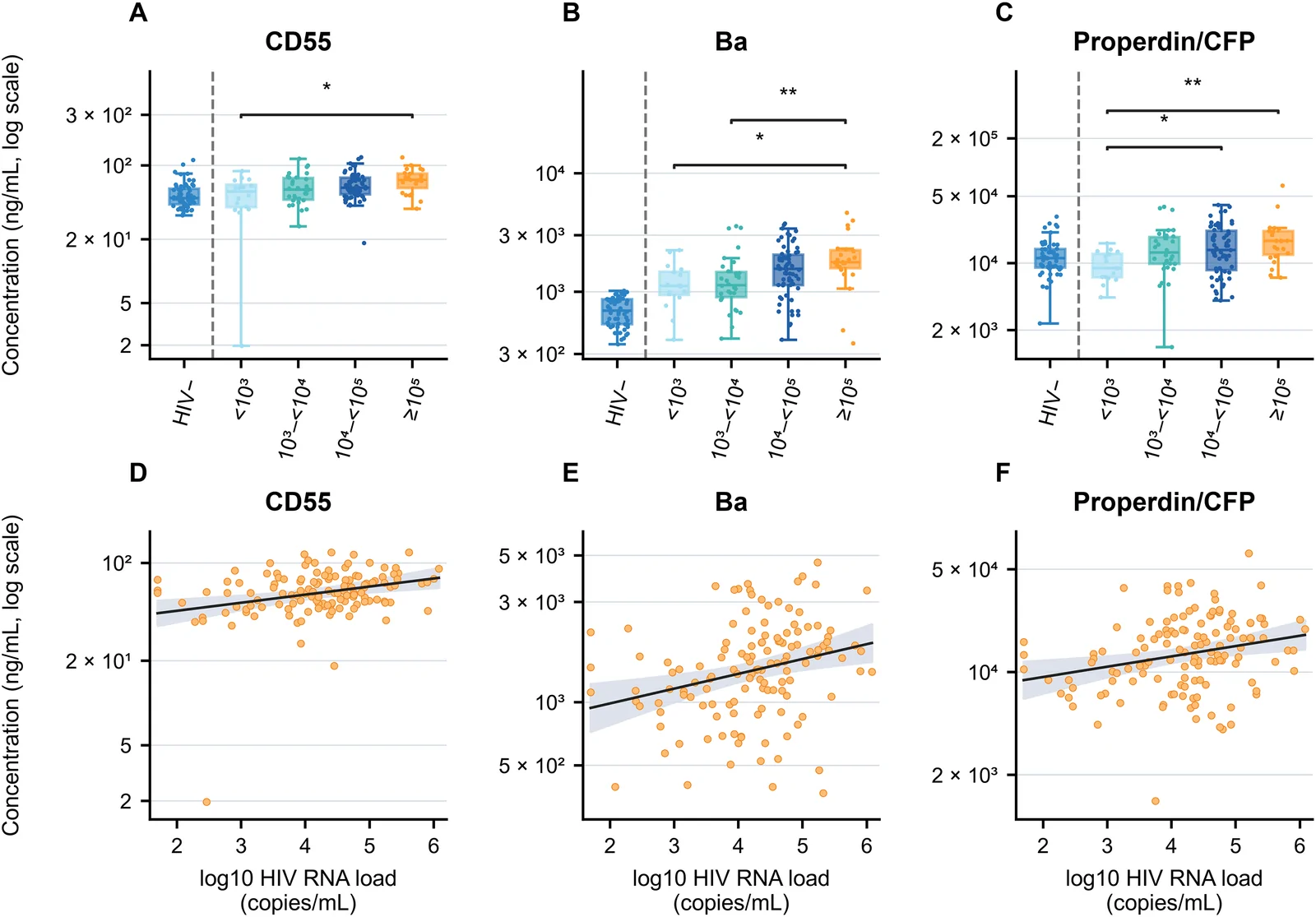
Plasma CD55, Ba, and properdin/CFP in relation to HIV RNA load. **(A–C)** show distributions in HIV-negative controls and four HIV-positive viral-load categories. A vertical dashed line separates controls, which are descriptive only and excluded from Kruskal-Wallis and Dunn tests. Boxes show medians and interquartile ranges; whiskers extend to the most extreme observations within 1.5 times the IQR of each quartile. Points represent individuals; y-axes are logarithmic. Brackets show significant Dunn comparisons after Holm correction across six comparisons within each marker (*adjusted P<0.05; **adjusted P<0.01). **(D–F)** show HIV-positive participants only. Lines and shaded 95% confidence bands represent unadjusted linear fits of log10 concentration on log10 HIV RNA load, back-transformed to concentration units. Adjusted selected-marker models are in **Supplementary Table S3**, and the extended eight-marker analyses are in **Supplementary Table S9**.

In models adjusted for age, sex, and CD4+ T-cell count, the coefficients relating log10 HIV RNA load to log10 CD55, Ba, and properdin/CFP concentrations were positive. For CD55, β was 0.067 per log10 viral-load unit (95% CI 0.008 to 0.125; nominal P = 0.0260). Estimates for Ba and properdin/CFP were 0.071 (95% CI 0.028 to 0.115; P = 0.0012) and 0.079 (95% CI 0.038 to 0.119; P = 0.0001), respectively (**Supplementary Table S3**). Unadjusted trends are shown in **Figures 2D–F**. In the extended eight-marker case-only analysis, Ba and properdin/CFP retained q<0.05, whereas CD55 did not (q=0.0693; **Supplementary Table S9**).

### 3.4 Age- and sex-adjusted associations between HIV status and complement markers

In age- and sex-adjusted log10-linear models, HIV status was positively associated with C3a, C5a, C1q, Ba, MASP-2, CD55, and properdin/CFP. Seven of the eight adjusted associations remained statistically significant after Benjamini-Hochberg correction (all q<0.05); CD59 did not. For plasma CD55, the adjusted estimate was β=0.070 (95% CI 0.021 to 0.119; P = 0.0054; q=0.0073), corresponding to an adjusted geometric mean ratio of 1.17 (95% CI 1.05 to 1.32), or approximately 17% higher plasma CD55 concentration in HIV-positive participants than in HIV-negative controls (**Figure 3**; **Supplementary Table S4**).

**Figure 3:**
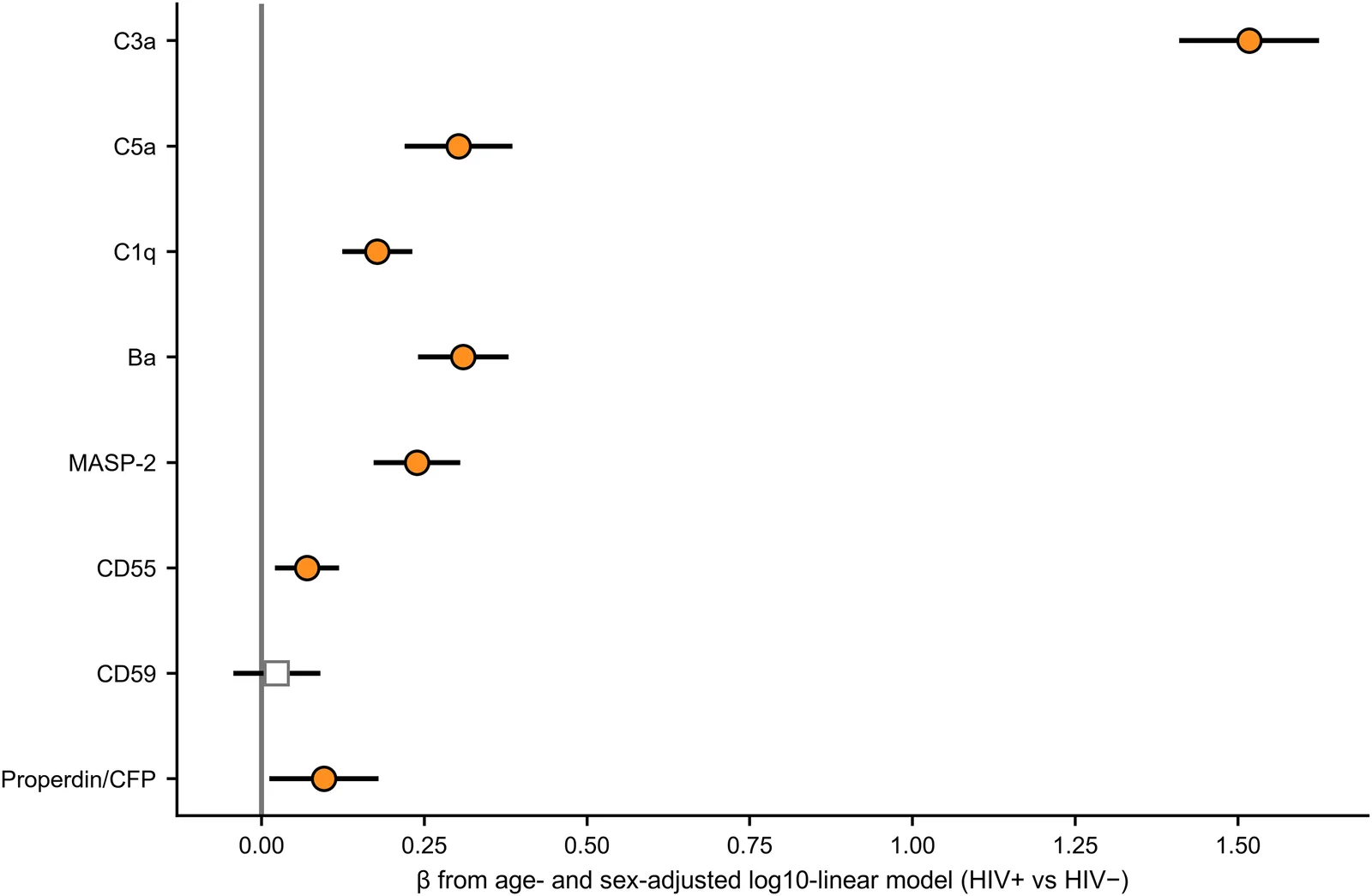
Age- and sex-adjusted associations of HIV status with plasma complement markers. Points represent regression coefficients (β) from log10-linear models comparing HIV-positive with HIV-negative participants; horizontal lines indicate 95% confidence intervals. Models used HC3 heteroskedasticity-robust standard errors. Filled circles indicate Benjamini-Hochberg q<0.05; the open square indicates q≥0.05. Numerical estimates and q values are reported in **Supplementary Table S4**.

### 3.5 Sensitivity analyses

The CD55 case-control estimates were positive in male-only and matched analyses, but both 95% confidence intervals included zero (**Supplementary Table S5**). Restriction to 120 adults without clinically identified coinfections yielded a similar adjusted CD55 case-control estimate (β=0.070, 95% CI 0.020 to 0.120; q=0.0083). Six of eight restricted case-control associations retained q<0.05; properdin/CFP did not (q=0.0516), nor did CD59. Within these 120 cases, the CD55–HIV RNA load estimate was positive (β=0.072, 95% CI 0.007 to 0.136; nominal P = 0.0294), but was not significant after eight-marker correction (q=0.0748; **Supplementary Tables S6**, **S9**). No marker differed significantly across CD4 strata (all omnibus P≥0.0719; **Supplementary Table S7**). With equal-sized viral-load quartiles, Ba and properdin/CFP differed across groups, whereas CD55 did not (P = 0.0645; **Supplementary Table S8**).

## 4 Discussion

Newly diagnosed, ART-naive HIV-positive adults showed differences in plasma complement markers relative to HIV-negative controls. The CD55 associations with HIV RNA load were nominally significant in continuous models in both the full and restricted case populations, but neither remained significant after eight-marker correction. The equal-sized quartile comparison was also not significant. The male-only and matched CD55 case-control estimates were imprecise. These findings warrant further investigation of plasma CD55 but do not establish a robust association with HIV RNA load.

Higher C3a and C5a concentrations are compatible with greater downstream complement activation, although their plasma levels also depend on generation, clearance, and specimen handling (6, 7, 10–12). C1q and MASP-2 concentrations describe pathway-associated protein abundance, whereas Ba reflects factor B cleavage. MASP-2 showed no relationship with viral load in either categorical or continuous analyses. The higher MASP-2 concentration in cases does not establish greater lectin-pathway activity, because neither MASP-2 enzymatic activity nor functional pathway activity was measured. These findings provide observations before ART initiation that complement earlier experimental and treated-cohort studies (10–13).

CD55 and CD59 act at different points in the complement cascade. CD55 accelerates C3/C5 convertase decay (3, 5, 16, 17), whereas membrane CD59 inhibits membrane attack complex formation (18, 19). Their plasma associations need not mirror their surface functions. Schmitz et al. showed that membrane CD55/CD59 expression and blockade influenced complement-mediated lysis of HIV-infected target cells (20). Saifuddin et al. demonstrated protection of HIV-1 particles by virion-associated CD55 and CD59 (8). These experiments concern cell- or virion-associated regulators, whereas our assays measured circulating immunoreactivity. The CD59 kit measures soluble CD59; neither assay established cell-surface abundance or biological activity. The assay did not distinguish freely soluble CD55 from membrane- or vesicle-associated CD55, nor did it determine whether shedding contributed to the measured concentration. Thus, elevated plasma CD55 cannot establish enhanced surface protection, virion incorporation, or shedding as its source.

Ba and properdin/CFP linked the plasma profile to alternative-pathway-related processes. Properdin stabilizes alternative-pathway convertases and promotes amplification (21, 22). Their viral-load associations were supported by continuous models and quartile comparisons, although the properdin/CFP case-control association in the restricted population was not significant after false discovery rate correction. Longitudinal measurements across ART initiation could distinguish changes accompanying viral suppression from persistent host inflammatory processes.

Complement can connect viral recognition with inflammatory signaling and coagulation, while host regulators can protect both host membranes and HIV particles (8, 23–26). The concentration of a circulating complement protein alone does not indicate whether its net effect favors host defense or viral protection. Studies in treated or virologically suppressed HIV cohorts also connect complement perturbations with non-AIDS comorbidities, inflammation, and coagulation abnormalities (27, 28). Our cross-sectional measurements do not identify which of these processes contributes to clinical outcomes or establish a rationale for complement-directed treatment.

The study combined pre-ART clinical measurements with a complement panel spanning activation fragments, pathway-associated proteins, and regulators. Both groups were recruited from the same testing setting and underwent the same plasma handling procedures.

Selection depended on clinical-data completeness and archived plasma availability, limiting generalizability beyond this hospital testing population. Age and sex imbalance leaves potential residual confounding despite adjustment and restricted analyses. Coinfection ascertainment relied on available clinical information rather than systematic screening. Hepatitis B/C results were unavailable, and infection activity and related treatment at sampling could not be established. The restricted analysis therefore cannot exclude residual effects of unrecognized infections or related treatment. Frozen storage and thawing may affect complement measurements, and shared handling does not exclude marker-specific preanalytical bias (29). Only EDTA plasma was studied, without CH50 or other functional complement assays. Together with the cross-sectional design and unresolved molecular forms of plasma regulators, these limitations restrict mechanistic and causal inference.

Overall, untreated HIV infection was associated with differences in circulating complement markers. The findings for plasma CD55 remain exploratory and require confirmation in independent cohorts. Longitudinal studies combining plasma measurements with cell-surface phenotyping and functional complement assays could clarify its relationship with HIV RNA load, molecular source, and biological significance.

## Data Availability

The original contributions presented in the study are included in the article/**Supplementary Material**. Further inquiries can be directed to the corresponding authors.
