# Supplementary material for: Plasma CD55 and HIV RNA load in newly diagnosed antiretroviral therapy-naive adults: an exploratory hospital-based case-control study
Source: Front Immunol. 2026 Oct 20;17:1981826. doi: 10.3389/fimmu.2026.1981826 (PMC13727742; doi:10.3389/fimmu.2026.1981826)
Supplement: Supplementary file 1 [file SupplementaryFile1.docx]

Supplementary Material

## Supplementary Figure S1 Participant selection

Participants were selected using available clinical information, sufficient archived EDTA plasma, and adult age. The restricted analysis excluded 12 cases with opportunistic infection (OI) documented at HIV diagnosis, including all three with tuberculosis (TB), and retained the same controls. No coinfection was clinically identified in the remaining 120 cases from the available reporting cards, medical records, and clinical history taking. Infection activity and related treatment at sampling were not established; HBV/HCV screening results were unavailable. Exclusion categories summarize eligibility and do not specify a chronological screening sequence.

## Supplementary Table S1 Complement assays and plasma dilution factors

| **Marker** | **Assay** | **Manufacturer** | **Catalog no.** | **HIV+ dilution** | **HIV− dilution** |
| --- | --- | --- | --- | --- | --- |
| C3a | MicroVue C3a Plus EIA | Quidel | A031 | 3000× or 6000× | 500× |
| C5a | MicroVue C5a EIA | Quidel | A021 | 50× | 20× |
| C1q | Human C1q ELISA Kit | Hycult Biotech | HK356 | 500× | 200× |
| Ba | MicroVue Ba EIA | Quidel | A033 | 2000× | 500× |
| MASP-2 | Human MASP-2 ELISA Kit | Hycult Biotech | HK326 | 50× | 50× |
| CD55 | Human CD55/DAF ELISA Kit | Sino Biological | KIT10101-1 | 20× | 20× |
| CD59 | Human sCD59 ELISA kit | Hycult Biotech | HK374-01 | 20× | 20× |
| Properdin/CFP | Human Properdin ELISA Kit | RayBiotech | ELH-PROPE-1 | 4000× | 4000× |

All readouts were OD 450 nm. Dilutions were selected using kit instructions and preliminary dilution testing where required. C3a plasma dilutions were 3000× or 6000× for HIV-positive samples and 500× for HIV-negative samples. Concentrations were corrected for the dilution factor applied to each sample. Concentrations throughout this supplement are in ng/mL, including CD55. Manufacturer locations are given in Section 2.5.

## Supplementary Table S2A Distributions across HIV RNA load categories

| **Marker** | **HIV− (n=59)** | **<10³ (n=16)** | **10³–<10⁴ (n=30)** | **10⁴–<10⁵ (n=63)** | **≥10⁵ (n=23)** | **KW P** |
| --- | --- | --- | --- | --- | --- | --- |
| C3a | 100.81 [80.14–138.68] | 1878.25 [1019.99–6315.87] | 3578.78 [1446.87–8345.42] | 6923.44 [4254.47–8903.22] | 3867.66 [1876.69–6853.73] | 0.0293 |
| C5a | 9.15 [6.39–11.63] | 17.99 [10.47–24.92] | 17.64 [13.55–25.42] | 17.03 [12.10–24.77] | 20.40 [12.41–27.90] | 0.8101 |
| C1q | 18526.89 [14811.02–21451.99] | 24249.61 [17795.34–38191.87] | 23771.23 [18061.65–29010.43] | 27633.28 [21039.98–40495.07] | 29808.87 [20240.02–44464.02] | 0.1977 |
| Ba | 697.40 [536.05–864.88] | 1125.09 [943.83–1484.49] | 1142.86 [906.00–1474.92] | 1566.41 [1142.94–2056.76] | 1779.17 [1580.40–2244.56] | 0.0024 |
| MASP-2 | 745.17 [616.43–996.40] | 1359.12 [1042.01–1799.18] | 1355.29 [1071.41–1888.98] | 1357.29 [1073.22–1869.65] | 1355.07 [1069.43–1727.98] | 0.9969 |
| CD55 | 49.07 [42.69–60.23] | 56.53 [40.23–65.88] | 58.83 [47.26–75.85] | 61.49 [53.20–76.26] | 72.77 [61.04–83.79] | 0.0170 |
| CD59 | 21.82 [18.38–26.50] | 24.87 [20.56–29.27] | 23.78 [21.43–27.98] | 25.65 [22.30–30.02] | 28.60 [24.69–37.83] | 0.0748 |
| Properdin/CFP | 11269.50 [9021.90–14034.94] | 8844.91 [7115.41–12553.95] | 12898.72 [9850.76–18755.10] | 13747.79 [8486.92–21808.54] | 17059.18 [12221.03–21630.00] | 0.0097 |

Values are median [Q1–Q3] in ng/mL. HIV RNA load categories are in copies/mL. Kruskal-Wallis (KW) P values are unadjusted for multiple testing and compare only the four HIV-positive categories. HIV-negative controls are descriptive and excluded from these tests.

## Supplementary Table S2B Dunn comparisons within significant omnibus marker tests

| **Marker** | **Category 1** | **Category 2** | **Dunn P** | **Holm P** |
| --- | --- | --- | --- | --- |
| C3a | <10³ | 10³–<10⁴ | 0.3158 | 0.9475 |
| C3a | <10³ | 10⁴–<10⁵ | 0.0112 | 0.0670 |
| C3a | <10³ | ≥10⁵ | 0.4395 | 0.9475 |
| C3a | 10³–<10⁴ | 10⁴–<10⁵ | 0.0715 | 0.2988 |
| C3a | 10³–<10⁴ | ≥10⁵ | 0.8319 | 0.9475 |
| C3a | 10⁴–<10⁵ | ≥10⁵ | 0.0598 | 0.2988 |
| Ba | <10³ | 10³–<10⁴ | 0.7325 | 0.7325 |
| Ba | <10³ | 10⁴–<10⁵ | 0.0339 | 0.1114 |
| Ba | <10³ | ≥10⁵ | 0.0026 | 0.0132 |
| Ba | 10³–<10⁴ | 10⁴–<10⁵ | 0.0278 | 0.1114 |
| Ba | 10³–<10⁴ | ≥10⁵ | 0.0016 | 0.0098 |
| Ba | 10⁴–<10⁵ | ≥10⁵ | 0.1141 | 0.2282 |
| CD55 | <10³ | 10³–<10⁴ | 0.3487 | 0.5058 |
| CD55 | <10³ | 10⁴–<10⁵ | 0.0521 | 0.2085 |
| CD55 | <10³ | ≥10⁵ | 0.0033 | 0.0201 |
| CD55 | 10³–<10⁴ | 10⁴–<10⁵ | 0.2529 | 0.5058 |
| CD55 | 10³–<10⁴ | ≥10⁵ | 0.0164 | 0.0821 |
| CD55 | 10⁴–<10⁵ | ≥10⁵ | 0.0913 | 0.2740 |
| Properdin/CFP | <10³ | 10³–<10⁴ | 0.0261 | 0.1043 |
| Properdin/CFP | <10³ | 10⁴–<10⁵ | 0.0057 | 0.0286 |
| Properdin/CFP | <10³ | ≥10⁵ | 0.0010 | 0.0057 |
| Properdin/CFP | 10³–<10⁴ | 10⁴–<10⁵ | 0.7023 | 0.7023 |
| Properdin/CFP | 10³–<10⁴ | ≥10⁵ | 0.1626 | 0.4878 |
| Properdin/CFP | 10⁴–<10⁵ | ≥10⁵ | 0.2148 | 0.4878 |

Dunn tests use pooled ranks and tie correction. Holm adjustment covers all six comparisons within each marker. Controls are excluded. Only markers with unadjusted omnibus P<0.05 proceed to post hoc inference.

## Supplementary Table S3 Selected marker models within HIV-positive participants

| **Marker** | **n** | **β (95% CI)** | **Nominal P** |
| --- | --- | --- | --- |
| Ba | 132 | 0.071 (0.028 to 0.115) | 0.0012 |
| CD55 | 132 | 0.067 (0.008 to 0.125) | 0.0260 |
| Properdin/CFP | 132 | 0.079 (0.038 to 0.119) | 0.0001 |

Outcome is log10 concentration; β is per one-unit increase in log10 HIV RNA load. Models adjust for age, sex, and CD4+ T-cell count and use HC3 standard errors. P values are nominal, not adjusted for multiple testing. The expanded eight-marker model family and its separate Benjamini-Hochberg correction are reported in Table S9.

## Supplementary Table S4 Age and sex adjusted case-control models

| **Marker** | **n** | **β (95% CI)** | **P** | **BH q** |
| --- | --- | --- | --- | --- |
| C3a | 191 | 1.517 (1.410 to 1.625) | <0.0001 | <0.0001 |
| C5a | 191 | 0.303 (0.220 to 0.386) | <0.0001 | <0.0001 |
| C1q | 191 | 0.178 (0.124 to 0.232) | <0.0001 | <0.0001 |
| Ba | 191 | 0.310 (0.240 to 0.379) | <0.0001 | <0.0001 |
| MASP-2 | 191 | 0.239 (0.172 to 0.305) | <0.0001 | <0.0001 |
| CD55 | 191 | 0.070 (0.021 to 0.119) | 0.0054 | 0.0073 |
| CD59 | 191 | 0.023 (-0.043 to 0.090) | 0.4923 | 0.4923 |
| Properdin/CFP | 191 | 0.096 (0.012 to 0.180) | 0.0250 | 0.0285 |

Outcome is log10 concentration. β compares HIV-positive with HIV-negative participants. Models use HC3 standard errors. Benjamini-Hochberg (BH) correction covers eight marker models.

## Supplementary Table S5 Demographic sensitivity analyses

## A Male-only analysis

| **Marker** | **n** | **β (95% CI)** | **Nominal P** |
| --- | --- | --- | --- |
| C3a | 159 | 1.552 (1.451 to 1.653) | <0.0001 |
| C5a | 159 | 0.312 (0.217 to 0.408) | <0.0001 |
| C1q | 159 | 0.151 (0.087 to 0.214) | <0.0001 |
| Ba | 159 | 0.277 (0.210 to 0.345) | <0.0001 |
| MASP-2 | 159 | 0.262 (0.192 to 0.332) | <0.0001 |
| CD55 | 159 | 0.049 (-0.007 to 0.105) | 0.0888 |
| CD59 | 159 | -0.008 (-0.083 to 0.066) | 0.8223 |
| Properdin/CFP | 159 | 0.063 (-0.009 to 0.135) | 0.0852 |

128 HIV-positive and 31 HIV-negative men. Models adjust for age and use HC3 standard errors.

## B Sex exact and age matched analysis

| **Marker** | **Pairs** | **Mean difference (95% CI)** | **Nominal P** |
| --- | --- | --- | --- |
| C3a | 32 | 1.453 (1.295 to 1.611) | <0.0001 |
| C5a | 32 | 0.315 (0.191 to 0.440) | <0.0001 |
| C1q | 32 | 0.187 (0.118 to 0.256) | <0.0001 |
| Ba | 32 | 0.288 (0.185 to 0.391) | <0.0001 |
| MASP-2 | 32 | 0.194 (0.117 to 0.272) | <0.0001 |
| CD55 | 32 | 0.062 (-0.006 to 0.131) | 0.0738 |
| CD59 | 32 | 0.027 (-0.047 to 0.101) | 0.4649 |
| Properdin/CFP | 32 | 0.081 (-0.042 to 0.205) | 0.1875 |

Optimal 1:1 matching without replacement, exact for sex within a five-year age caliper. The procedure maximized matched pairs and then minimized total absolute age distance. A linear-sum assignment was solved separately within each sex, with controls as rows and cases plus unmatched dummy assignments as columns. Eligible assignments had absolute age difference as cost; dummy assignments cost 10,000 and ineligible assignments cost 100,000,000. Original input order was retained. Multiple equally optimal pair sets may exist; the final pair list defines the reported analysis. The resulting 32 pairs comprised 28 male and four female pairs, with a maximum absolute age difference of four years and a total absolute age difference of 21 years. Estimates are mean within-pair HIV-positive minus HIV-negative log10 concentrations, with t-based intervals and one-sample t tests. P values in both panels are exploratory and unadjusted for multiplicity.

## Supplementary Table S6 Restricted population without clinically identified coinfections

120 HIV-positive adults without clinically identified coinfections, based on available reporting cards, medical records, and clinical history taking, and the same 59 HIV-negative controls. The restricted population was obtained by excluding the 12 cases with OI documented at HIV diagnosis, including all three tuberculosis cases. This clinical classification does not establish a negative systematic infection screen; HBV/HCV results and infection activity and related treatment at sampling were unavailable.

## A Unadjusted concentrations

| **Marker** | **HIV+ median [IQR]** | **HIV− median [IQR]** | **MWU P** |
| --- | --- | --- | --- |
| C3a | 5346.42 [1772.05–8483.62] | 100.81 [80.14–138.68] | <0.0001 |
| C5a | 16.96 [12.08–24.91] | 9.15 [6.39–11.63] | <0.0001 |
| C1q | 25908.57 [19679.36–37076.05] | 18526.89 [14811.02–21451.99] | <0.0001 |
| Ba | 1427.53 [990.32–1928.63] | 697.40 [536.05–864.88] | <0.0001 |
| MASP-2 | 1356.18 [1045.94–1837.19] | 745.17 [616.43–996.40] | <0.0001 |
| CD55 | 61.14 [50.73–76.58] | 49.07 [42.69–60.23] | <0.0001 |
| CD59 | 25.13 [21.55–29.74] | 21.82 [18.38–26.50] | 0.0029 |
| Properdin/CFP | 13488.42 [8753.11–19822.03] | 11269.50 [9021.90–14034.94] | 0.0589 |

Concentrations are ng/mL. Mann-Whitney U P values are unadjusted.

## B Adjusted case-control models

| **Marker** | **n** | **β (95% CI)** | **P** | **BH q** |
| --- | --- | --- | --- | --- |
| C3a | 179 | 1.501 (1.387 to 1.615) | <0.0001 | <0.0001 |
| C5a | 179 | 0.281 (0.192 to 0.369) | <0.0001 | <0.0001 |
| C1q | 179 | 0.179 (0.119 to 0.239) | <0.0001 | <0.0001 |
| Ba | 179 | 0.297 (0.228 to 0.366) | <0.0001 | <0.0001 |
| MASP-2 | 179 | 0.245 (0.175 to 0.315) | <0.0001 | <0.0001 |
| CD55 | 179 | 0.070 (0.020 to 0.120) | 0.0062 | 0.0083 |
| CD59 | 179 | 0.016 (-0.055 to 0.087) | 0.6575 | 0.6575 |
| Properdin/CFP | 179 | 0.092 (0.002 to 0.182) | 0.0452 | 0.0516 |

Age- and sex-adjusted HC3 models. BH correction covers these eight restricted case-control models, independently of the main models.

## Supplementary Table S6 continued

## C Selected marker case-only models

| **Marker** | **n** | **β (95% CI)** | **Nominal P** |
| --- | --- | --- | --- |
| Ba | 120 | 0.081 (0.032 to 0.130) | 0.0012 |
| CD55 | 120 | 0.072 (0.007 to 0.136) | 0.0294 |
| Properdin/CFP | 120 | 0.090 (0.044 to 0.136) | 0.0001 |

Age-, sex-, and CD4-adjusted HC3 models in the 120 restricted cases. Expanded models for all eight markers and BH q values are in Table S9.

## D Restricted viral-load distributions

| **Marker** | **<10³ (n=15)** | **10³–<10⁴ (n=29)** | **10⁴–<10⁵ (n=58)** | **≥10⁵ (n=18)** | **KW P** |
| --- | --- | --- | --- | --- | --- |
| C3a | 1701.40 [1019.99–6380.88] | 2937.03 [1347.81–8182.16] | 6922.90 [4237.50–9010.59] | 4072.36 [1927.46–6983.32] | 0.0322 |
| C5a | 16.98 [10.45–24.47] | 16.94 [13.43–24.94] | 15.28 [11.97–24.35] | 20.26 [12.64–28.53] | 0.6873 |
| C1q | 21858.22 [17493.28–38291.74] | 23160.09 [17885.18–28698.37] | 25475.27 [20847.34–38718.83] | 33401.67 [24947.95–45022.03] | 0.0847 |
| Ba | 1134.92 [928.69–1505.14] | 1126.76 [885.85–1433.94] | 1484.53 [1138.04–1955.69] | 1863.45 [1686.47–2308.75] | 0.0012 |
| MASP-2 | 1284.02 [1042.01–1706.29] | 1314.67 [1058.98–1917.90] | 1355.50 [1064.03–1892.88] | 1437.40 [1207.46–1931.61] | 0.8744 |
| CD55 | 51.96 [39.65–67.08] | 58.76 [46.83–73.96] | 61.06 [53.10–76.55] | 73.42 [67.75–84.13] | 0.0174 |
| CD59 | 25.31 [20.43–29.34] | 23.67 [21.17–27.60] | 25.13 [22.01–28.21] | 30.05 [25.44–38.27] | 0.0447 |
| Properdin/CFP | 8831.51 [7059.71–11994.89] | 12122.41 [9839.11–18129.01] | 14726.23 [8347.02–22000.10] | 18378.86 [13014.16–22494.64] | 0.0039 |

Values are median [Q1–Q3] in ng/mL. Unadjusted omnibus tests compare four HIV-positive categories only.

## Supplementary Table S6 continued

## E Restricted Dunn comparisons

| **Marker** | **Category 1** | **Category 2** | **Dunn P** | **Holm P** |
| --- | --- | --- | --- | --- |
| C3a | <10³ | 10³–<10⁴ | 0.3547 | 1.0000 |
| C3a | <10³ | 10⁴–<10⁵ | 0.0107 | 0.0641 |
| C3a | <10³ | ≥10⁵ | 0.3530 | 1.0000 |
| C3a | 10³–<10⁴ | 10⁴–<10⁵ | 0.0503 | 0.2517 |
| C3a | 10³–<10⁴ | ≥10⁵ | 0.9194 | 1.0000 |
| C3a | 10⁴–<10⁵ | ≥10⁵ | 0.1243 | 0.4971 |
| Ba | <10³ | 10³–<10⁴ | 0.9476 | 0.9476 |
| Ba | <10³ | 10⁴–<10⁵ | 0.0804 | 0.1608 |
| Ba | <10³ | ≥10⁵ | 0.0017 | 0.0087 |
| Ba | 10³–<10⁴ | 10⁴–<10⁵ | 0.0328 | 0.1173 |
| Ba | 10³–<10⁴ | ≥10⁵ | 0.0003 | 0.0021 |
| Ba | 10⁴–<10⁵ | ≥10⁵ | 0.0293 | 0.1173 |
| CD55 | <10³ | 10³–<10⁴ | 0.3429 | 0.4227 |
| CD55 | <10³ | 10⁴–<10⁵ | 0.0431 | 0.1724 |
| CD55 | <10³ | ≥10⁵ | 0.0036 | 0.0215 |
| CD55 | 10³–<10⁴ | 10⁴–<10⁵ | 0.2113 | 0.4227 |
| CD55 | 10³–<10⁴ | ≥10⁵ | 0.0169 | 0.0846 |
| CD55 | 10⁴–<10⁵ | ≥10⁵ | 0.1090 | 0.3270 |
| CD59 | <10³ | 10³–<10⁴ | 0.7967 | 1.0000 |
| CD59 | <10³ | 10⁴–<10⁵ | 0.7539 | 1.0000 |
| CD59 | <10³ | ≥10⁵ | 0.0375 | 0.1499 |
| CD59 | 10³–<10⁴ | 10⁴–<10⁵ | 0.4475 | 1.0000 |
| CD59 | 10³–<10⁴ | ≥10⁵ | 0.0070 | 0.0420 |
| CD59 | 10⁴–<10⁵ | ≥10⁵ | 0.0183 | 0.0916 |
| Properdin/CFP | <10³ | 10³–<10⁴ | 0.0322 | 0.1289 |
| Properdin/CFP | <10³ | 10⁴–<10⁵ | 0.0036 | 0.0180 |
| Properdin/CFP | <10³ | ≥10⁵ | 0.0004 | 0.0024 |
| Properdin/CFP | 10³–<10⁴ | 10⁴–<10⁵ | 0.4761 | 0.4761 |
| Properdin/CFP | 10³–<10⁴ | ≥10⁵ | 0.0632 | 0.1895 |
| Properdin/CFP | 10⁴–<10⁵ | ≥10⁵ | 0.1427 | 0.2854 |

Six pairwise comparisons per marker, conditional on omnibus P<0.05. Holm adjustment is within marker.

## Supplementary Table S7 Complement markers across CD4 strata

| **Marker** | **<200 (n=37)** | **200–500 (n=75)** | **>500 (n=20)** | **KW P** |
| --- | --- | --- | --- | --- |
| C3a | 6884.96 [2172.54–9640.62] | 4827.15 [1635.59–7094.37] | 7282.80 [4030.05–8865.80] | 0.0719 |
| C5a | 23.72 [13.43–28.28] | 16.98 [11.87–24.80] | 14.86 [12.31–23.55] | 0.2024 |
| C1q | 29114.44 [20591.55–34476.28] | 26281.07 [20060.14–35838.35] | 24624.94 [18433.08–43405.21] | 0.8396 |
| Ba | 1635.10 [1243.65–2169.88] | 1363.52 [971.85–1897.03] | 1553.43 [1081.60–2219.46] | 0.1977 |
| MASP-2 | 1337.25 [1128.57–1713.90] | 1395.90 [1037.47–1886.40] | 1395.95 [1084.04–1825.13] | 0.9450 |
| CD55 | 62.48 [48.65–80.53] | 61.10 [51.26–74.93] | 63.59 [51.80–77.23] | 0.7177 |
| CD59 | 25.65 [23.29–36.09] | 25.11 [21.42–29.15] | 25.91 [20.80–29.27] | 0.2155 |
| Properdin/CFP | 13827.24 [9021.45–19675.18] | 13671.61 [8101.11–19874.56] | 12342.25 [10362.68–16448.65] | 0.8933 |

CD4+ T-cell strata are cells/μL; concentrations are median [Q1–Q3], ng/mL. Tests include 132 HIV-positive participants only. Omnibus P values are unadjusted. No marker passed the P<0.05 gate, so no post hoc significance is claimed.

## Supplementary Table S8 Equal-sized HIV RNA load quartiles

Exploratory quartile groups contain 33 HIV-positive participants each. Internal log10 HIV RNA load boundaries are 3.744239, 4.341895, 4.763239. Concentrations are median [Q1–Q3], ng/mL. Q1 is the lowest viral-load quartile.

| **Marker** | **Q1** | **Q2** | **Q3** | **Q4** | **KW P** |
| --- | --- | --- | --- | --- | --- |
| C3a | 2515.06 [1347.81–8399.84] | 5307.49 [1744.05–7901.78] | 6448.43 [4827.15–9169.42] | 6051.10 [1928.88–8193.58] | 0.1149 |
| C5a | 16.98 [12.48–24.97] | 17.03 [13.05–20.57] | 15.21 [12.34–24.88] | 20.97 [12.06–28.32] | 0.6906 |
| C1q | 24382.37 [18591.05–33670.25] | 24947.03 [18026.71–32436.69] | 28564.40 [21514.03–43915.21] | 27633.28 [20743.62–44169.82] | 0.3203 |
| Ba | 1126.76 [958.97–1433.94] | 1428.50 [977.25–1927.23] | 1532.74 [1136.52–1951.49] | 1860.73 [1536.48–2315.83] | 0.0006 |
| MASP-2 | 1308.47 [1042.01–1855.76] | 1395.90 [1032.93–1917.03] | 1357.29 [1096.07–1713.90] | 1355.07 [1128.57–1763.97] | 0.9860 |
| CD55 | 60.39 [46.60–72.02] | 58.91 [51.30–73.96] | 61.11 [51.89–76.84] | 71.28 [54.81–80.53] | 0.0645 |
| CD59 | 24.44 [20.74–28.90] | 24.19 [21.68–32.10] | 25.85 [22.95–30.34] | 26.68 [24.05–32.20] | 0.1511 |
| Properdin/CFP | 10554.95 [8684.98–13675.04] | 15632.81 [7995.10–22089.57] | 13747.79 [11104.06–21425.42] | 17059.18 [11774.32–21578.48] | 0.0376 |

## Post hoc comparisons

| **Marker** | **Quartile 1** | **Quartile 2** | **Dunn P** | **Holm P** |
| --- | --- | --- | --- | --- |
| Ba | Q1 | Q2 | 0.0296 | 0.1480 |
| Ba | Q1 | Q3 | 0.0349 | 0.1480 |
| Ba | Q1 | Q4 | <0.0001 | 0.0002 |
| Ba | Q2 | Q3 | 0.9474 | 0.9474 |
| Ba | Q2 | Q4 | 0.0473 | 0.1480 |
| Ba | Q3 | Q4 | 0.0404 | 0.1480 |
| Properdin/CFP | Q1 | Q2 | 0.0339 | 0.1697 |
| Properdin/CFP | Q1 | Q3 | 0.0378 | 0.1697 |
| Properdin/CFP | Q1 | Q4 | 0.0068 | 0.0406 |
| Properdin/CFP | Q2 | Q3 | 0.9653 | 1.0000 |
| Properdin/CFP | Q2 | Q4 | 0.5570 | 1.0000 |
| Properdin/CFP | Q3 | Q4 | 0.5282 | 1.0000 |

Omnibus P values are unadjusted. Post hoc tests are reported only after omnibus P<0.05; Holm correction covers six comparisons within each marker. CD55 omnibus P=0.0645, so no CD55 pairwise significance is claimed.

## Supplementary Table S9 Extended exploratory case-only models for all eight markers

## A All HIV-positive adults

| **Marker** | **n** | **β (95% CI)** | **P** | **BH q** |
| --- | --- | --- | --- | --- |
| C3a | 132 | 0.083 (-0.018 to 0.184) | 0.1088 | 0.1741 |
| C5a | 132 | -0.014 (-0.063 to 0.035) | 0.5729 | 0.6547 |
| C1q | 132 | 0.027 (-0.013 to 0.067) | 0.1826 | 0.2435 |
| Ba | 132 | 0.071 (0.028 to 0.115) | 0.0012 | 0.0048 |
| MASP-2 | 132 | -0.000 (-0.030 to 0.029) | 0.9883 | 0.9883 |
| CD55 | 132 | 0.067 (0.008 to 0.125) | 0.0260 | 0.0693 |
| CD59 | 132 | 0.018 (-0.002 to 0.038) | 0.0738 | 0.1477 |
| Properdin/CFP | 132 | 0.079 (0.038 to 0.119) | 0.0001 | 0.0011 |

## B Adults without clinically identified coinfections

| **Marker** | **n** | **β (95% CI)** | **P** | **BH q** |
| --- | --- | --- | --- | --- |
| C3a | 120 | 0.086 (-0.024 to 0.196) | 0.1238 | 0.1650 |
| C5a | 120 | -0.013 (-0.067 to 0.042) | 0.6505 | 0.6505 |
| C1q | 120 | 0.039 (-0.006 to 0.083) | 0.0874 | 0.1398 |
| Ba | 120 | 0.081 (0.032 to 0.130) | 0.0012 | 0.0049 |
| MASP-2 | 120 | 0.014 (-0.017 to 0.044) | 0.3758 | 0.4295 |
| CD55 | 120 | 0.072 (0.007 to 0.136) | 0.0294 | 0.0748 |
| CD59 | 120 | 0.023 (0.001 to 0.045) | 0.0374 | 0.0748 |
| Properdin/CFP | 120 | 0.090 (0.044 to 0.136) | 0.0001 | 0.0009 |

β is the change in log10 marker concentration per one-unit increase in log10 HIV RNA load. All models adjust for age, sex, and CD4+ T-cell count using HC3 standard errors. BH correction is applied across eight markers separately in each population. These expanded analyses are exploratory. The selected-marker estimates in Tables S3 and S6 are repeated here solely to show the complete correction family.
